# A transducible nuclear/nucleolar protein, mLLP, regulates neuronal morphogenesis and synaptic transmission

**DOI:** 10.1038/srep22892

**Published:** 2016-03-10

**Authors:** Nam-Kyung Yu, Hyoung F. Kim, Jaehoon Shim, Somi Kim, Dae Won Kim, Chuljung Kwak, Su-Eon Sim, Jun-Hyeok Choi, Seohee Ahn, Juyoun Yoo, Sun-Lim Choi, Deok-Jin Jang, Chae-Seok Lim, Yong-Seok Lee, Chulhun Kang, Soo Young Choi, Bong-Kiun Kaang

**Affiliations:** 1 Department of Biological Sciences, College of Natural Sciences, Seoul National University, Seoul, South Korea; 2 Department of Biomedical Engineering, Sungkyunkwan University, Suwon, South Korea; 3 Department of Biomedical Science and Research Institute for Bioscience and Biotechnology, Hallym University, Chunchon, South Korea; 4 Applied Biology, College of Ecological Environment, Kyungpook National University, 386, Gajang-dong, Sang-Ju, South Korea; 5 Department of Life Science, Chung-Ang University, Seoul, South Korea; 6 Graduate School of East-West Medical Science, Kyung Hee University, Kyungkee-do, South Korea

## Abstract

Cell-permeable proteins are emerging as unconventional regulators of signal transduction and providing a potential for therapeutic applications. However, only a few of them are identified and studied in detail. We identify a novel cell-permeable protein, mouse LLP homolog (mLLP), and uncover its roles in regulating neural development. We found that mLLP is strongly expressed in developing nervous system and that mLLP knockdown or overexpression during maturation of cultured neurons affected the neuronal growth and synaptic transmission. Interestingly, extracellular addition of mLLP protein enhanced dendritic arborization, demonstrating the non-cell-autonomous effect of mLLP. Moreover, mLLP interacts with CCCTC-binding factor (CTCF) as well as transcriptional machineries and modulates gene expression involved in neuronal growth. Together, these results illustrate the characteristics and roles of previously unknown cell-permeable protein mLLP in modulating neural development.

Neural development is the fundamental process for constructing the brain circuits underlying animal behavior, cognition, and emotion^1^. Various molecular processes have to be dynamically and meticulously orchestrated for normal neuronal morphogenesis and wiring of neural circuits during development^2^,^3^. An intriguing molecular pathway emerging in the field of neural development is the non-cell-autonomous action of homeodomain transcription factors^4^. Traditionally, intercellular signal transmission has been known to be mediated either by ligands that bind to membrane receptors or by the steroid hormones that translocate into cells. In recent reports, however, homeoproteins such as Engrailed-1/2, OTX2, PAX6, VAX1, and HOXD1 can penetrate into cells and directly exert their effects in neural development^4^,^5^,^6^. These cell-permeable proteins not only reveal a novel mechanism of intercellular signal transmission for neural development but also have therapeutic implications in the treatment of neurodegenerative disorders^7^. In addition, the domains of those proteins conferring the cell permeability can be used for transporting other molecules into cells^8^. However, very few of these molecules have been identified to date^4^,^9^.

Our group previously identified *Aplysia* LAPS18-Like Protein (ApLLP) as a nuclear/nucleolar protein that is induced by neuronal activity and upregulates C/EBP transcription, thereby enhancing synaptic plasticity^10^,^11^. Another study prior to ours showed that Learning-Associated Protein of Slug with 18 kDa (LAPS18), the LLP homolog in *Limax*, is upregulated in neurons after associative learning, and that LAPS18 addition to the extracellular space affects cell migration^12^. LLP homologs (LLPH) such as ApLLP and LAPS18 are largely conserved throughout animal species and might play important roles in the nervous system. Currently, however, there are no reports regarding the function of LLPH in vertebrate species.

ApLLP has been shown to be an intrinsically disordered protein (IDP), a protein without an ordered three-dimensional structure, in a previous structural analysis^13^,^14^,^15^. In contrast to the traditional view that structure of a protein is crucial for its function, numerous IDPs or unstructured regions of proteins have been identified as key players in biological processes such as signaling^16^, transcription^17^, and chromatin remodeling^18^. Due to their flexibility, IDPs can often bind to a variety of molecules and act as molecular hubs of protein-protein interaction networks^19^. Therefore, the molecular or cellular contexts and interaction partners could be important determinants for the functional modes of IDPs. Mammalian LLPH might be intrinsically disordered as ApLLP, and its interaction with other molecules might be important for its functions. Deregulated IDP expression levels are often linked with disease^19^,^20^, calling for the investigation of IDPs and their interacting molecules.

In this study, we identified mouse LLPH (mLLP) as a cell-permeable nuclear/nucleolar protein that is intrinsically disordered. To explore its roles in neural development, we overexpressed or knocked down mLLP in cultured hippocampal neurons during morphogenesis, which revealed that mLLP regulates dendritic and spine growth and synaptic transmission. Intriguingly, extracellular addition of mLLP produced a non-cell-autonomous effect on dendritic arborization. We also identified proteins interacting with mLLP relevant for regulating neuronal growth. Together, our results reveal a novel mechanism involving mLLP in modulating neuronal development.

## Results

### mLLP is a nuclear/nucleolar protein with cell permeability

**Figure 1:**
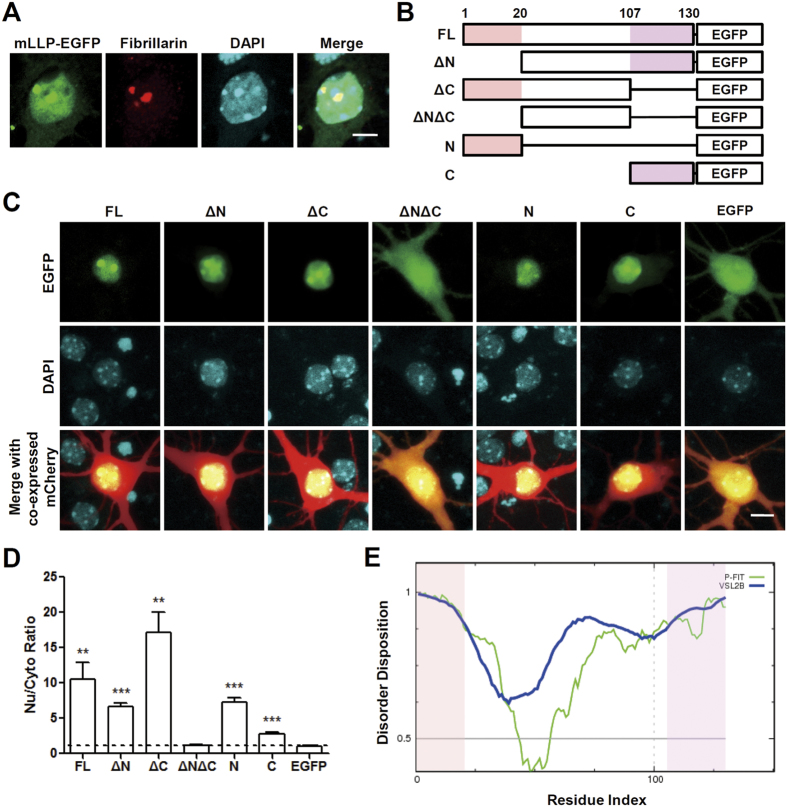
Subcellular localization and intrinsic disorder of mLLP protein. (**A**) Nuclear/nucleolar localization of mLLP. Anti-fibrillarin (nucleolar marker) immunocytochemistry of neurons transfected with plasmid encoding mLLP-EGFP. Scale bar, 5 μm. (**B**) Diagram representing the various deletion mutants of mLLP fused to EGFP. mLLP full-length (FL) sequence, mutant constructs (ΔN or ΔC) absent of N-terminal (1–20) or C-terminal (107–130) parts, or either N- or C-terminal sequences were cloned into the pEGFP-N1 multiple cloning site. (**C**) Representative fluorescence microscopy images of neurons expressing EGFP fused with mLLP deletion mutants. The construct expressing mCherry driven by neuron-specific CaMKII promoter was co-transfected. Scale bar, 10 μm. (**D**) Quantitative measurement of the ratio of nuclear/cytosolic EGFP signals in (**C**). EGFP signal intensity normalized by that of mCherry was compared between in the nucleus and in cytosol of the somatic region. The dotted line indicates the value 1, which means no differential localization in the nucleus and cytosol. One sample t-test (hypothetical value 1), **p < 0.01, ***p < 0.001. Data are represented as mean ± standard error mean (SEM). (**E**) Disorder analysis of mLLP using two different methods VSL2B (blue) and PONDR-FIT (P-FIT, green) in DisProt. The residues with value exceeding 0.5 are considered disordered.

LLP homologs are evolutionarily well conserved. Forty-six of the 130 amino acids in mLLP (~35.4%) are identical to ApLLP, and 110 amino acids (~84.6%) are identical to human LLPH (hLLP) (Supplementary Fig. 1). The polybasic nature of N- and C-terminal regions commonly appears in LLPH of various species. Similar to ApLLP^21^, mLLP fused to enhanced green fluorescent protein (mLLP-EGFP) was preferentially localized to the nucleus (Fig. 1A,B), showing an 10.6-fold enrichment in the nucleus relative to the cytosol (Fig. 1C). mLLP-EGFP formed intense sub-nuclear spots that were immunostained with fibrillarin, a nucleolar marker (Fig. 1A). To map which part of mLLP contributes to its nuclear localization, we expressed EGFP fused with various deletion mutants of mLLP (Fig. 1B). N-EGFP, C-EGFP, ΔN-EGFP and ΔC-EGFP were enriched in the nucleus relative to the cytosol, demonstrating that either N- or C-terminal region of mLLP (amino acids 1–20 and 107–130, respectively) is sufficient for nuclear localization (Fig. 1B,D). This preferential localization in the nucleus was not observed in ΔNΔC-EGFP, showing that at least one of N- or C-region is necessary for nuclear localization of mLLP. However, our quantification data revealed that the effects of nuclear localization by the two regions are not simply additive. Deletion of C-region from the full length (FL) mLLP interestingly augmented the nuclear localization (comparing the ΔC vs. FL) whereas deletion of C-region in the absence of N-region (comparing the ΔN vs. ΔNΔC) abolished the nuclear localization, suggesting that N- and C-regions may interact each other (Fig. 1C,D). Moreover, N-EGFP tended to localize to the nucleus more efficiently than C-EGFP, indicating that N-region seems to play a more dominant role in the nuclear localization of mLLP (Fig. 1D).

Since mLLP does not contain any well-identified functional domains and ApLLP was revealed as an IDP^13^,^14^,^15^, we analyzed its amino acid sequence using the database of protein disorder DisProt (http://www.disprot.org/metapredictor.php)^13^,^22^,^23^. The amino acid residues of mLLP were mostly scored as values >0.5, the threshold to predict the disorder probability (Fig. 1E). N- and C-regions containing nuclear localization signals were the most highly unstructured, which is consistent with other reports arguing that nuclear localization signals are often found in disordered sequences^24^,^25^,^26^.

**Figure 2:**
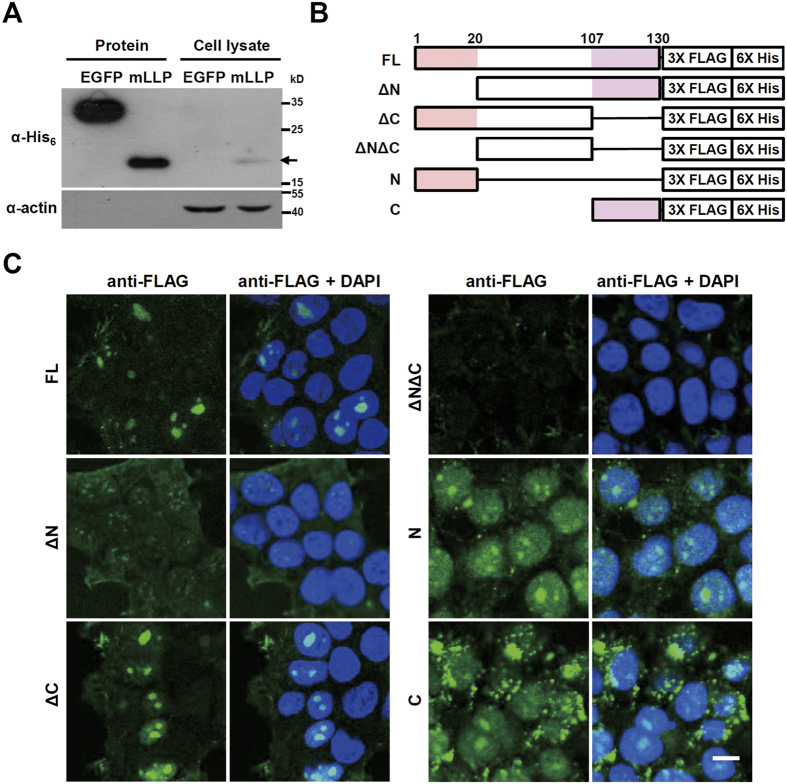
mLLP protein is cell-permeable. (**A**) Immunoblot analysis of HEK293T cell lysates 1 h after mLLP protein addition to the culture medium. mLLP-His_6_ protein was detected in the cell lysate, but EGFP protein used as a control was not. Loading controls (actin) were detected on the same blot. Samples are derived from the same experiment and processed in parallel. (**B**) Diagram representing the various deletion mutants of mLLP fused to 3XFLAG and hexahistidine tag. (**C**) N- and C-terminal regions of mLLP mediate protein transduction into cells. Immunofluorescent images of HEK293T cells treated with proteins produced from each fusion constructs. Scale bar, 10 μm.

As the intrinsic disorder and polybasic nature of N- and C-terminal regions of mLLP are shared with many cell penetrating peptides^27^, we asked whether mLLP could be internalized into cells. We added purified hexahistidine-tagged mLLP (mLLP-His_6_) to the culture media of HEK293T cells, followed by trypsinization to remove proteins attached to the cell surface, and then harvested the cells. mLLP-His_6_ was detected in the cell lysates, indicating that mLLP may be cell-permeable (Fig. 2A). Cell penetration of mLLP was confirmed by immunocytochemistry (Fig. 2B,C), in which 3xFLAG-tagged mLLP added to the culture media was detected in the nuclei of cells. To determine the domains responsible for the cell penetration, we examined the internalization of various mLLP deletion mutants fused to the 3xFLAG tag (Fig. 2B). Similar to the results of nuclear localization, the mutant with both N- and C-terminal regions deleted (ΔNΔC) was not detected inside the cells, suggesting the necessity of N- or C-terminal regions for cell permeability (Fig. 2C). However, mutants with one of either N- or C-terminal region deleted (ΔN or ΔC) were detected in the nuclei (Fig. 2C), suggesting the N- or the C-terminal domain is sufficient for internalization and subsequent nuclear localization of the protein. Furthermore, we found that mLLP is internalized into neurons when it is injected into mouse brain (Supplementary Fig. 2), confirming the cell-permeability of the protein and suggesting the potential application of mLLP for *in vivo* study. Collectively, these data reveal that mLLP is a cell-permeable nuclear/nucleolar protein, and that the N- or C-terminal domain confers this permeability.

### mLLP can modulate dendritic growth in both cell-autonomous and non-cell-autonomous manners

**Figure 3:**
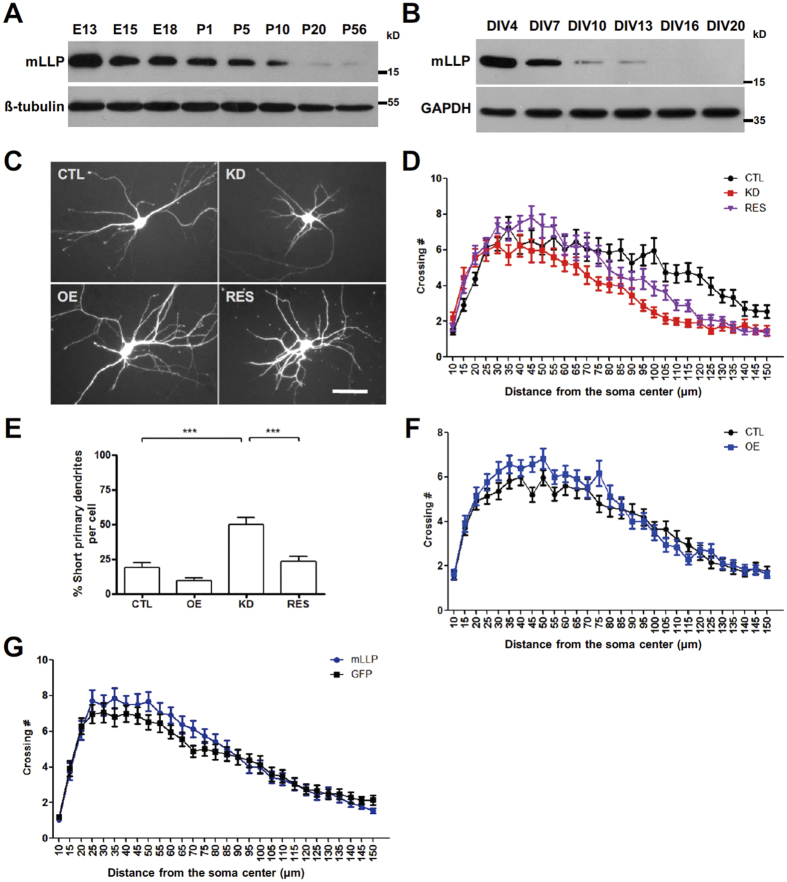
Developmental expression pattern of mLLP and its roles in the dendritic morphogenesis. (**A**) Western blot analysis of the whole brain lysates of mice from embryonic stages to adult. mLLP protein level in the brain is gradually decreased during development. Loading controls (β-tubulin) were detected on the same blot. Samples are derived from the same experiment and processed in parallel. (**B**) Western blot analysis of mLLP expression level in dissociated mouse hippocampal neuron cultures with time. Loading controls (GAPDH) were detected on the same blot. Samples are derived from the same experiment and processed in parallel. (**C**) Representative microscopy images of neurons at DIV 6 transfected differently into following groups at DIV 3: control (CTL, scrambled shRNA + control vector), mLLP overexpression (OE, scrambled shRNA + shRNA-resistant mLLP), mLLP knockdown (KD, shRNA against mLLP + control vector), or mLLP rescue (RES, shRNA against mLLP + shRNA-resistant mLLP). Scale bar, 50 μm. (**D**) Sholl analysis of neurons transfected with constructs for KD, RES, or CTL (two-way ANOVA, n = 24–25 cells per group, interaction ***p < 0.0001, group effect ***p < 0.0001). (**E**) The ratio of primary dendrites shorter than 30 μm (one-way ANOVA and post hoc Tukey’s multiple comparison test, *p < 0.05, ***p < 0.001, n = 29–30 cells per group). (**F**) Sholl analysis of mLLP overexpressing neurons or control neurons (n = 30 cells per group, two-way ANOVA, group effect **p = 0.0048). (**G**) Sholl analysis at DIV 6 after daily treatment with mLLP (1μg/mL) or control GFP protein for 3 days (n = 29 cells per group). Extracellular addition of mLLP protein increased the dendritic arborization compared with control. (two-way ANOVA, group effect *p = 0.0338). All the data are represented as mean ± SEM.

To investigate the role of mLLP in the nervous system, we first examined the expression pattern of mLLP using an antibody that we generated (Supplementary Fig. 3A,B) and found that mLLP is expressed in various tissues, including the brain (Supplementary Fig. 3C). We also assessed the developmental expression pattern of mLLP in whole brain lysates, which clearly showed its stronger expression in the early developmental stages and a sharp decline until adulthood (Fig. 3A). Moreover, the same expression pattern was reproduced in the primary hippocampal neuron culture, which undergoes morphological development for about two weeks after being plated onto the culture dish (Fig. 3B).

The stronger expression of mLLP in neurons during earlier developmental stages led us to hypothesize that mLLP might be an important regulator of neural development. Using the cultured hippocampal neurons, we examined whether mLLP is required for neuronal morphogenesis by knockdown of mLLP. We transfected neurons with plasmids encoding an shRNA against mLLP or a control scrambled shRNA at days-in-vitro (DIV) 3 and analyzed the dendritic structures of transfected neurons at DIV 6 (Fig. 3C). Sholl analysis revealed that dendritic growth was disrupted in mLLP knockdown (KD) group compared with the control group expressing the scrambled shRNA (CTL) and that the impairment was diminished by co-transfection of shRNA-resistant mLLP (RES) (Fig. 3D and Supplementary Fig. 3A). Transfection of another shRNA against mLLP (KD #2) also led to the abnormal dendritic morphology, supporting the role of mLLP in dendritic morphogenesis (Supplementary Fig. 4A,B). Knockdown of mLLP also increased the proportion of short primary neurites (<30 μm) per cell, which was recovered to the control level by co-transfecting the shRNA-resistant mLLP (Fig. 3E). Moreover, overexpression of mLLP conversely promoted the dendritic growth in Sholl analysis (Fig. 3F). We next examined whether increasing intracellular mLLP by administering cell permeable mLLP can affect the dendritic growth of neurons in a non-cell-autonomous manner. After daily treatment of mLLP protein into the culture medium for three days, neurons were fixed and subjected to Sholl analysis, which showed that mLLP administration enhanced the dendritic arborization compared with addition of GFP (Fig. 3G). Together, these results suggest that mLLP can regulate dendritic growth during neuronal maturation.

### mLLP regulates the density of dendritic protrusions and synaptic transmission

**Figure 4:**
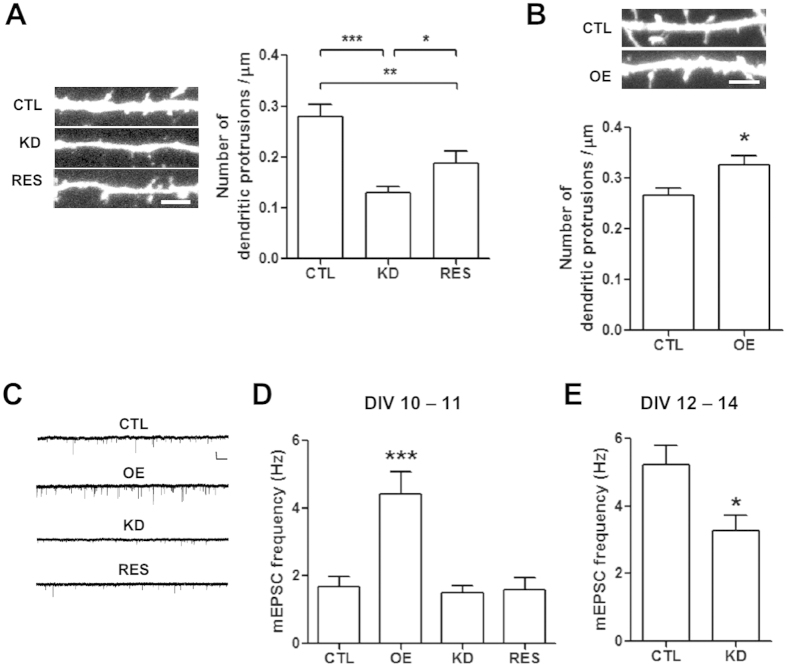
mLLP modulates the density of dendritic protrusions and synaptic transmission. (**A**) (Left) Representative confocal microscopic images of dendritic fragments of neurons at DIV 10 after transfection at DIV 6. Scale bars, 5 μm. (Right) Bar graphs representing the density of dendritic protrusions in transfected neurons. The density of dendritic protrusions in mLLP KD neurons was reduced compared with control, which was partially recovered in RES (one-way ANOVA and *post hoc* Newmann Keuls multiple comparison test: *p < 0.05, **p < 0.01, ***p < 0.001, n = 21 cells per group). (**B**) mLLP overexpression increased the density of dendritic protrusions (unpaired t-test: *p = 0.0148, n = 29–30 cells). (**C**) Sample recording traces of mEPSC (n = 12–13 cells). Scale bar, x = 500 ms, y = 20 pA. (**D**) mEPSC frequency is increased by mLLP overexpression at DIV 10–11 (one-way ANOVA with *post hoc* Newmann Keuls multiple comparison test: OE VS CTL, ***p < 0.001; KD VS CTL, KD VS RES, CTL VS RES, not significant, n = 10–13 cells). (**E**) mEPSC frequency is reduced in mLLP KD neurons at DIV 12–14 (unpaired t-test: *p = 0.0124, n = 14–18 cells). All data are represented as mean ± SEM.

Because dendritic and spine growth often involve common molecular mechanisms^28^, we sought to examine whether mLLP could also regulate spinogenesis (Fig. 4A). We transfected the plasmids that are designed to regulate mLLP expression levels in cultured neurons at DIV 6 and assessed the dendritic protrusions at DIV 10. At this time, many of the dendritic protrusions appeared to be filopodia or immature spines in our culture condition. We considered these protrusions as the premature structures that may grow as functional spines and counted these dendritic protrusions in transfected neurons. The density of dendritic protrusions was significantly reduced in KD neurons and was partially recovered by shRNA-resistant mLLP (Fig. 4A). In line with this result, overexpression of mLLP increased the density of dendritic protrusions (Fig. 4B), further supporting that mLLP may modulate spinogenesis.

We further examined whether mLLP could regulate synaptic transmission by measuring the miniature excitatory postsynaptic current (mEPSC) (Fig. 4C). mEPSC frequency at DIV 10–11 was increased in the mLLP OE neurons, but it was not affected in the KD neurons (Fig. 4D). Furthermore, at DIV 10–11, mLLP over-expression seemed to have the relatively large effect on mEPSC frequency compared with the effect on the number of dendritic protrusions (Fig. 4B,D), implying that over-expression of mLLP may facilitate the maturation of the spines, leading to an increase in the proportion of functional spines. At later stages of DIV 12–14 (Fig. 4E), mEPSC frequency in control neurons became higher than at DIV 10–11, indicating that more functional synapses had been generated. In this condition, mEPSC frequency was significantly reduced in KD neurons (Fig. 4E), consistent with the reduced number of filopodia and spines in KD neurons at DIV 10–11. mEPSC frequency was also reduced in KD #2 neurons (Supplementary Fig. 4C). mEPSC amplitude, however, did not seem to be consistently affected by mLLP knockdown or overexpression despite some reduction in KD neurons (Supplementary Fig. 5). Taken together, these results suggest that mLLP may affect the synaptic transmission possibly through regulating spinogenesis.

### Upstream and downstream mechanisms of mLLP in regulating neural development

**Figure 5:**
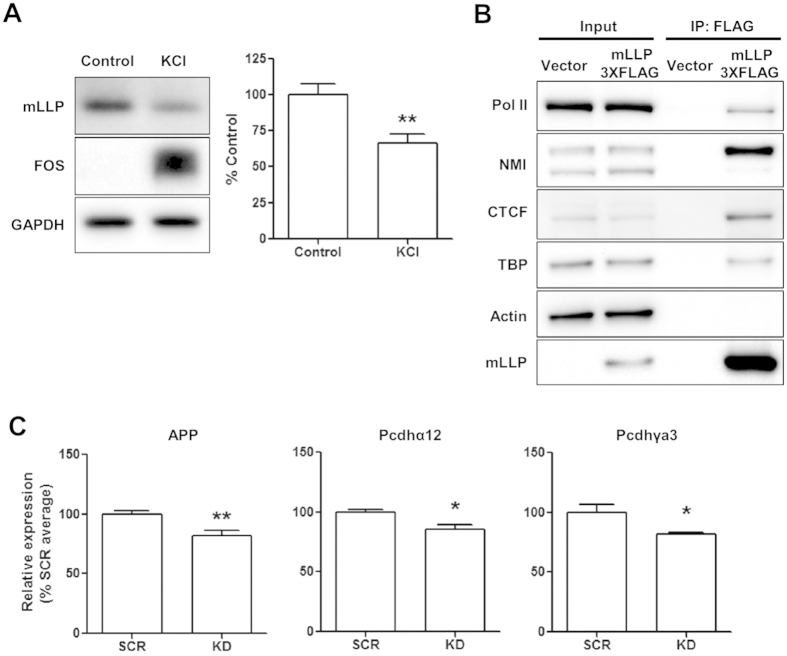
Upstream and downstream mechanisms of mLLP acting on neural development. (**A**) Neuronal activation by KCl treatment for 2 h at DIV 7 decreases mLLP protein expression level (right panel: quantification data of mLLP/GAPDH, n = 8 per group, unpaired t-test: **p = 0.0044). Loading controls (GAPDH) were detected on the same blot. Samples from the two groups were processed in the same experiment in parallel. Extended blot images including this data are presented in Supplementary Fig. 6B. (**B**) Nuclear extracts from HEK293T cells transfected with 3XFLAG-tagged mLLP or control plasmid were immunoprecipitated with anti-FLAG affinity gel and immunoblotted with antibodies against each protein indicated. All data are derived from the same blot, and the samples were processed in the same experiment in parallel. Extended blot images are presented in Supplementary Fig. 8. (**C**) qRT-PCR analysis of a subset of known CTCF target genes after mLLP knockdown in dissociated hippocampal neuron cultures at DIV 6 (unpaired t-test: **p < 0.01, *p < 0.05, n = 4 per group). All data are represented as mean ± SEM.

Neuronal development and synaptogenesis are known to be regulated by neuronal activity. We examined whether neuronal activity regulates mLLP expression level. Activation of neurons by increasing the extracellular potassium concentration (40 mM KCl) for a prolonged time (2–5 h) in cultured neurons reduced the mLLP protein level whereas the stimulus remarkably up-regulated Fos, which is a well-known marker for neuronal activity (Fig. 5A and Supplementary Fig. 6A,B). This stimulus also down-regulated the mLLP mRNA level (Supplementary Fig. 6C). We also found that mLLP is a substrate of calcium-dependent protease calpain that plays critical roles in synaptic plasticity^29^ (Supplementary Fig. 6D) in line with the notion that IDPs are generally unstable and vulnerable to proteolysis^30^. These results suggest that sustained neuronal activity can down-regulate mLLP expression at multiple steps, which might be important for homeostatic plasticity considering the impact of mLLP on neuronal growth and synaptic transmission.

Although mLLP is localized to the nucleolus, its knockdown or overexpression in neurons did not significantly affect the nucleolar size or number (Supplementary Fig. 7A,B). To investigate the molecular functions of mLLP, we screened the proteins interacting with mLLP in the nucleus by performing mass spectrometry following immunoprecipitation using the nuclear lysates of NIH3T3 cells overexpressing mLLP. Mass spectrometry showed that nuclear myosin I β (NMI) interacts with mLLP, which was confirmed by western blotting after co-immunoprecipitation (Fig. 5B). In addition, as NMI is involved in transcriptional regulation, we assessed whether TATA-binding protein (TBP) and RNA polymerase II (Pol II), the components of transcriptional machineries, are also co-immunoprecipitated with mLLP (Fig. 5B). Albeit weaker than NMI, TBP and Pol II were repeatedly detected in the proteins pulled down with mLLP. Actin, which was detected in a substantial amount in the input nuclear lysates, was not present in the proteins co-immunoprecipitated with mLLP, indicating that specific proteins are pulled down and detected in our assay. These results showing the interaction of mLLP with transcriptional machinery suggest the possibility that mLLP may be involved in transcriptional regulation.

Interestingly, in a recent high-throughput interactome study, hLLP was shown to interact with CTCF^31^, which is the only protein known to bind to insulator sequences in vertebrates and plays essential roles in 3D chromatin organization and transcriptional regulation^32^,^33^. CTCF plays essential roles in postnatal brain development^28^ and mutations in this protein are found in patients with cognitive deficits^34^. However, the molecular mechanism of how CTCF regulates gene expression in the nervous system is not well understood. We confirmed that mLLP also interacts with CTCF by co-immunoprecipitation assay (Fig. 5B). These results suggest that mLLP could act as a transcriptional regulator by interacting with transcription machinery or other transcription factors such as CTCF.

Since mLLP knockdown leads to a similar impairment in dendritic growth, spine density and synaptic transmission similar to CTCF deletion^28^, we assumed that mLLP might modulate CTCF-mediated regulation of genes involved in neural development. We performed qRT-PCR analysis of mRNA expression of amyloid precursor protein (APP) and some protocadherin genes (Pcdhs), which have been reported to be under the control of CTCF in neurons and are implicated in neuronal morphogenesis^35^,^36^,^37^,^38^. Neurons infected with adeno-associated virus (AAV) expressing shRNA against mLLP showed reduced expression level of APP, Pcdhα12, and Pcdhγa3 compared with scrambled shRNA control (Fig. 5C). Using another sequence of shRNA, we found the similar effect of mLLP knockdown on the regulation of these genes (Supplementary Fig. 4D). These results suggest that mLLP and CTCF might work together, at least partially, to control gene expression required for neural development.

## Discussion

In the present study, we report the characteristics and physiological roles of the mammalian homolog of ApLLP, which we call mLLP. mLLP modulates dendritic morphogenesis and synaptic transmission in cultured neurons. Moreover, mLLP is a cell-permeable nuclear/nucleolar protein and intrinsically disordered, opening a new window for extending the knowledge of these unique molecular features.

We observed that administration of mLLP protein into the extracellular culture medium enhanced the dendritic growth of cultured neurons, which is similar to the previous study showing that the addition of Engrailed-2 protein affects axonal outgrowth^5^. However, the effect of a cell-permeable protein on dendritic arborization has not been reported before. Interestingly, there are evidences that LLP homologs (LLPHs) can bind polyadenylated RNA molecules^39^ and ribosomal proteins (http://thebiogrid.org/), implying that internalized mLLP might modulate translation as Engrailed-2^5^. Our result raises an intriguing question whether endogenous mLLP can be secreted and transferred to other cells to regulate dendritic growth^40^. Notably, LAPS18, the LLP homolog in the slug, was initially reported as a secreted protein^12^, implicating that mLLP might also be secreted in certain condition. Further investigations are needed to examine the possibility of non-cell-autonomous action of endogenous mLLP. In addition, the effect of mLLP in cultured neurons brings up a question whether mLLP protein administration can be effective *in vivo*. We showed that mLLP protein can be delivered into the brain cells (Supplementary Fig. 2), but its physiological consequence was not assessed here. Future studies might find the potential for therapeutic use of mLLP^8^.

Considering the high sequence similarity of mLLP and hLLP (Supplementary Fig. 1), we may assume that they have common molecular features and physiological roles. Indeed, the interaction of CTCF and hLLP was initially found in human proteome^31^. In addition, we found that the intrinsic disorder (Supplementary Fig. 9C) and cell penetrating property (Supplementary Fig. 9D) are also observed in hLLP. According to the RNA sequencing database^41^, the mRNA expression of LLPH in primates also shows a decreasing pattern during development (Supplementary Fig. 9A,B). The similar molecular characteristics and developmental expression patterns of hLLP and mLLP suggest that hLLP could also play an important role in human brain development and cognitive function.

Intrinsic disorder of LLP homologs suggests that the tight control of its expression would be important for normal cellular functions^19^,^20^. Consistent with the notion, our results showed that knockdown or overexpression of mLLP altered neuronal development and functions *in vivo*. As a highly disordered protein as a whole, LLPH might interact with various molecules, acting as a linker in protein interaction network^15^. This emphasizes the importance of identifying the interacting molecules of LLPH. We particularly looked into the interaction of LLPH with CTCF, which has been extensively studied as the only documented insulator-binding protein in vertebrates and a multifunctional DNA-binding zinc-finger protein^42^. The variety of its functions also comes from its interaction with various other proteins^43^, but the binding partners or molecular processes underlying the role of CTCF in neural development have not been reported. Our results show that mLLP interacts with CTCF and that mLLP knockdown impairs neuronal morphogenesis and synaptic transmission, similar to deletion of CTCF^28^. Some CTCF target genes that are important for neural development were downregulated after mLLP knockdown. These data suggest that CTCF, at least in part, might function by interacting with mLLP in coordinating dendritic growth and spinogenesis. However, the exact molecular mechanisms involved in the function of LLPH and its interaction with CTCF are mostly unknown, thus remain to be studied in the future. It would also be interesting to examine the functions of LLPH in other tissues, given that mLLP is ubiquitously expressed and that CTCF plays crucial roles in diverse tissues.

In summary, we present a molecular characterization of a nuclear/nucleolar protein called mLLP as a cell-permeable and intrinsically disordered protein and show its roles in regulating neuronal morphogenesis and synaptic transmission. Our study unveils a novel mechanism involved in neural development.

## Methods

### Animals

All experimental procedures were approved by the Institutional Animal Care and Use Committee of Seoul National University and in accordance with their guidelines. All the mice used were C57BL6/N from KOATEK.

### DNA constructs

mLLP cDNA was cloned by PCR with hippocampal cDNA of C57BL/6 as a template. It was again subcloned into the vector for expression in the mammalian cells (pcDNA3.1(+)-mLLP) or in the E.coli with hexahistidine tag attached at C-terminal (pET21a-mLLP). shRNA target sequences were GCCGAGAAGAGAAAGAAGA (shmLLP, KD) and GAAAGAACGAGGAGAGACA (scrambled, SCR). The shRNA oligos were designed and inserted into the pSuper-GFP.neo vector following the product manual. shRNA-resistant mLLP sequence was generated by recombinant PCR and inserted into the pcDNA3.1(+) vector.

### Dissociated mouse hippocampal neuron culture

Hippocampi were dissected from E17 embryos and dissociated mechanically after trypsin treatment. Approximately 40,000 cells/cm^2^ were plated onto poly-D-lysine-coated plastic culture dishes or cover slips. After a 3–4 h recovery in the media composed of MEM-EBBS with 2 mM glutamine, 10% FBS, 0.45% glucose, 0.11 mg/mL sodium pyruvate, and penicillin/streptomycin, cells were maintained in Neurobasal medium supplemented with B27, glutamax, and penicillin/streptomycin. Transfection was performed using Lipofectamine2000 (Invitrogen) according to the manual provided by manufacturer.

### Immunoblotting

Scraped cultured cells or mouse whole brains (3–7 animals/age group) were snap-frozen in liquid nitrogen and later lysed in RIPA buffer. The same amount of lysates were subjected to SDS-PAGE and transferred onto nitrocellulose membranes. Membranes were incubated with primary antibodies (mLLP antibody, affinity purified from serum produced from the rabbit injected with purified mLLP protein, 1:250, anti-GAPDH, Ambion, 1:1000,000) overnight at ~4 °C and then with HRP-conjugated secondary antibodies for 30 min at room temperature. Chemiluminescent signals were detected with HRP substrates (Millipore, WBKLS0100) using ChemiDoc system or developing solutions, and band intensities were quantified using ImageJ software.

### Sholl analysis

Neurons were transfected with pcDNA3.1(+) or pcDNA3.1(+)-mLLP at 3 DIV. For visualization, GFP-expressing pSuper plasmid was co-transfected. At 6 DIV, neurons were fixed with 4% paraformaldehyde/4% sucrose in PBS. GFP images were taken under a fluorescence microscope and dendrites were traced using the NeuronJ plugin of ImageJ. The traces were subjected to Sholl analysis using the ImageJ plugin. Protein-treated neurons were processed by the same method except that the transfection of the pcDNA3.1(+) plasmid was omitted. Two or three independent cultures were used for each experiment.

### Spine analysis

Transfected neurons were imaged with a 40X objective lens and Z-stack mode in a confocal microscope (LSM700). Viewed in Zen (Zeiss) software, numbers of dendritic protrusions were counted for three 50 μm dendritic segments per neuron, and 9–10 neurons per cover slip were analyzed. This was repeated for three independent cultures.

### Electrophysiological recordings

For whole-cell patch-clamp recordings, cultured hippocampal neurons (DIV 10–11 and DIV 12–14) were patched and kept in −70 mV using a Multiclamp 700B amplifier and pClamp 10.2 software (Molecular Devices). Ten min after rupturing, data were collected for 5 min and digitized at 10 kHz with a 2 kHz lowpass filter using Digidata 1440 16-bit A/D converter (Axon instruments). The recording pipettes (3 ~ 5 MΩ) were pulled with P-1000 (Sutter instrument) with three step protocol and filled with internal solution containing 145 K-Gluconate, 5 mM NaCl, 0.2 mM EGTA, 10 mM HEPES, 2 mM MgATP, 0.1 mM Na3GTP, 1 mM MgCl_2_ (pH 7.2 with KOH, 280 ~ 290 mOsm). The bath solution contained 124 mM NaCl, 2.5 mM KCl, 1 mM NaH_2_PO_4_, 25 mM NaHCO_3_, 10 mM Glucose, 2 mM CaCl_2_, 2 mM MgSO_4_. For mEPSC recording, Picrotoxin (100 μM) and Tetrodotoxin (1 μM) were added to the bath solution to block GABAA receptor mediated current and to block evoked synaptic responses, respectively. The bath solution was oxygenated with 5% Co_2_, 95% O_2_ mix gas and perfused 1 ~ 2 ml/min at 25 ~ 26 °C. Series resistances were carefully monitored and recordings were not used if it changed significantly (>20%) or reached 15 MΩ. Cells that required more than 200 pA of hold current to maintain −70 mV were excluded from the dataset. Data were analyzed using Clampfit 10.4 (Molecular Devices) with template match threshold of 4. To analyze data with template match function in the program, template was created by averaging about 50 traces in one of the recording files analyzed. All the recording files in the experiment were analyzed with the same template.

### Co-immunoprecipitation

Epitope-tagged mLLP or control vector-transfected cells were lysed with hypotonic buffer (10 mM HEPES [pH 7.9], 10 mM KCl, 0.1 mM EDTA, 0.1 mM EGTA, 1 mM DTT) to isolate nuclei. Nuclei were lysed with lysis buffer (20 mM HEPES [pH7.9], 400 mM KCl, 1 mM EDTA, 1 mM EGTA, 1 mM DTT). Nuclear lysates were dialyzed with IP buffer (25 mM Tris [pH 7.4], 150 mM NaCl, 0.1% Triton X-100). Nuclear lysates were incubated with anti-FLAG agarose beads (Sigma) o/n at 4 °C. They were washed with IP buffer with 0.1 mM EDTA and 0.2% Triton X-100 and then buffer without Triton X-100. Immunoprecipitated proteins were eluted with 2X SDS sample buffer at 85 °C for 3 min, and subjected to immunoblotting after gel electrophoresis using Bolt 4–12% Bis-Tris Plus Gels.

### Immunocytochemistry

Cells were fixed with 4% paraformaldehyde/4% sucrose in PBS. These cells were permeabilized with 0.1% Triton X-100, 0.1% BSA in PBS (PBT) and then blocked with 0.08% Triton X-100, 2% BSA in PBS. Cells were incubated with primary antibodies (anti-FLAG [Sigma], anti-fibrillarin [Covance], anti-GFP [Neuromab]) o/n at 4 °C and washed with PBT. Next, they were incubated with secondary antibodies conjugated with fluorescence dyes were at room temperature for 2 h and washed with PBT. Samples were mounted on Vectashield with DAPI (VectorLab) and imaged using confocal microscopy (LSM700).

### AAV generation

The expression cassettes of shRNA from pSuper-GFP.neo-shmLLP or scrambled were inserted into AAV2 vector. AAV was generated by transfection of these vectors encoding transgenes and plasmids for AAV1 packaging into HEK293T cells^44^. Following the purification by iodixanol gradient method from the supernatant media of transfected cells, the solution was concentrated and exchanged to PBS using Amicon Ultra-15. The titer of virus was measured as viral genome (vg) copy number calculated in comparison with the copy number of plasmids by real time PCR.

### Statistics

All statistical analyses were performed using Graphpad Prism software. When there are two experimental groups, we performed the Student’s t-test. For more than two experimental groups, we performed ANOVA test, and in case there is a significant difference among the groups, multiple comparison tests between the groups were conducted. The data of one neuron in the KD group and one neuron in the KD #2 group in mEPSC recording at DIV 12–14 were excluded as outliers by performing the Grubb’s test. The numbers of biological replicates or animal numbers and p-values are written for each data in the figure legends.

## Additional Information

**How to cite this article**: Yu, N.-K. *et al.* A transducible nuclear/nucleolar protein, mLLP, regulates neuronal morphogenesis and synaptic transmission. *Sci. Rep.* **6**, 22892; doi: 10.1038/srep22892 (2016).

## Supplementary Material

Supplementary Information
