# Supplementary material for: A transducible nuclear/nucleolar protein, mLLP, regulates neuronal morphogenesis and synaptic transmission
Source: Sci Rep. 2016 Mar 10;6:22892. doi: 10.1038/srep22892 (PMC4790632; doi:10.1038/srep22892)
Supplement: Supplementary Information [file srep22892-s1.doc]

**Supplementary Information**

**A transducible nuclear/nucleolar protein, mLLP, regulates neuronal morphogenesis and synaptic transmission**

Nam-Kyung Yu^1^, Hyoung F. Kim^1,2^, Jaehoon Shim^1^, Somi Kim^1^, Dae Won Kim^3^, Chuljung Kwak^1^, Su-Eon Sim^1^, Jun-Hyeok Choi^1^, Seohee Ahn^1^, Juyoun Yoo^1^, Sun-Lim Choi^1^, Deok-Jin Jang^4^, Chae-Seok Lim^1^, Yong-Seok Lee^5^, Chulhun Kang^6^, Soo Young Choi^3^ and Bong-Kiun Kaang^1*^

^1^ Department of Biological Sciences, College of Natural Sciences, Seoul National University, Seoul, South Korea

^2^ Department of Biomedical Engineering, Sungkyunkwan University, Suwon, South Korea

^3^ Department of Biomedical Science and Research Institute for Bioscience and Biotechnology, Hallym University, Chunchon, South Korea

^4^ Applied Biology, College of Ecological Environment, Kyungpook National University, 386, Gajang-dong, Sang-Ju, South Korea.

^5^ Department of Life Science, Chung-Ang University, Seoul, South Korea.

^6^ Department of Neuroscience, Graduate School of East-West Medical Science, Kyunghee University, Kyungkee-do, South Korea

*Correspondence: kaang@snu.ac.kr

**Supplementary Figure 1. The amino acid sequence alignment of LLPH of various animal species**

Basic amino acids are indicated as magenta, acidic as blue, polar as green, and small or hydrophobic as red colored letters. Boxed areas represent the N- and C-terminal regions containing the nuclear localization signals. These regions are highly enriched with arginine and lysine. The polybasic nature of N- and C-terminal portions is a common feature of LLPH, which implies that the overall charge distribution could be important for the molecular function of LLPH.


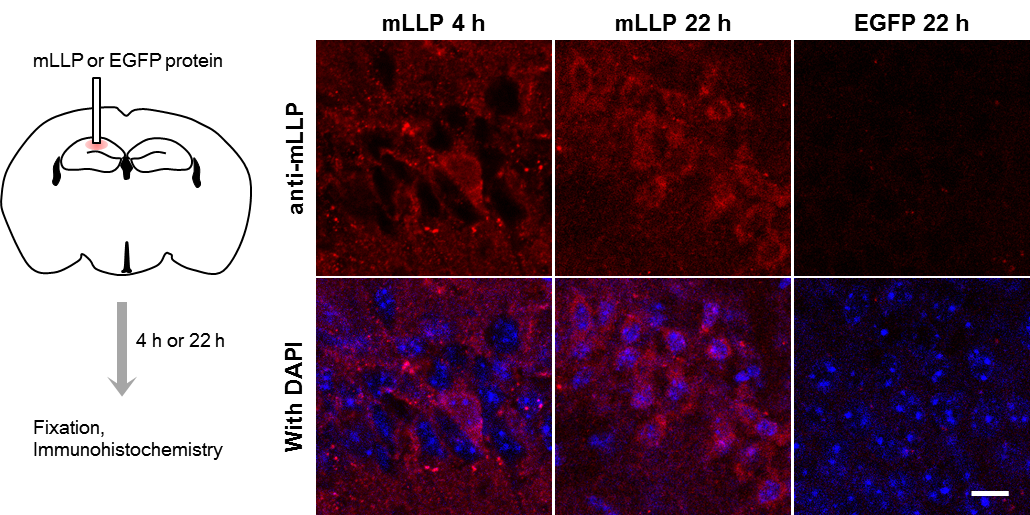


**Supplementary Figure 2. *In vivo* delivery of mLLP protein**

Anti-mLLP immunohistochemistry at 4 or 22 h after stereotactic injection of mLLP or 22 h after injection of GFP protein into the mouse hippocampal CA1. The amount of injected protein was 1.7 μg in DPBS. At 4 h after the mLLP protein injection, mLLP protein mostly appeared to be near the cell surface and only a few cells internalized the mLLP. At 22 h after the injection, most of the cells in the injection site have internalized the mLLP protein. The signals look stronger in the cytosol than in the nucleus at this time point in our experimental condition using the mouse brain.


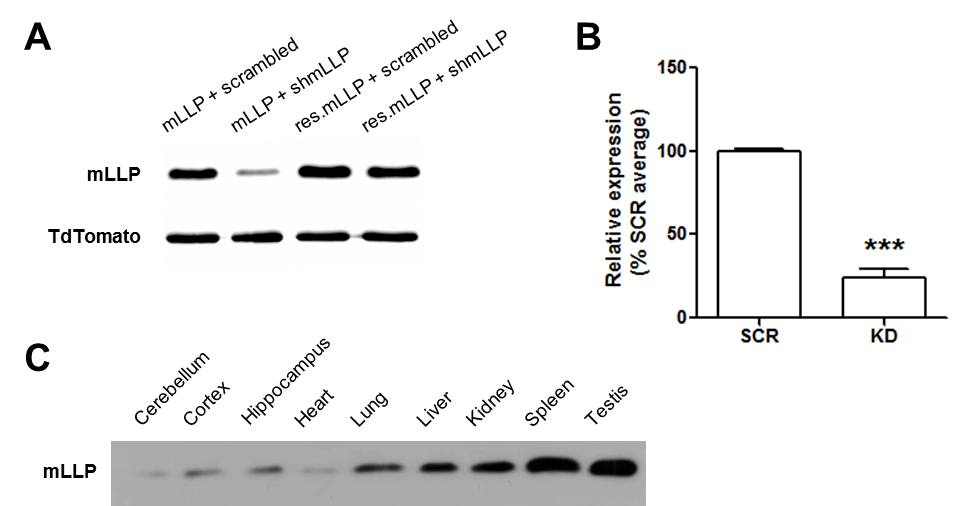


**Supplementary Figure 3. Tissue expression pattern of mLLP protein**

(A) Verification of mLLP antibody and shRNA-resistant mLLP construct (res.mLLP). Overexpressed mLLP protein in HEK293T cells was detected by the purified antibody as a band around 18 kD, which was diminished by co-expression with shRNA against mLLP (shmLLP). shRNA-resistant mLLP (res.mLLP) expression was not affected by shmLLP. To confirm the equal transfection efficiency, TdTomato was co-expressed. shRNA expression substantially reduced the wild-type mLLP expression by ~ 77.4 %.

(B) The expression of shRNA against mLLP using adeno-associated viral vector in cultured neurons for 7 – 10 days reduced the level of mLLP mRNA by ~76.0 %. Unpaired t-test: ***p < 0.0001, n = 4 – 5 per group.

(C) mLLP protein is ubiquitously expressed in various organ tissues.


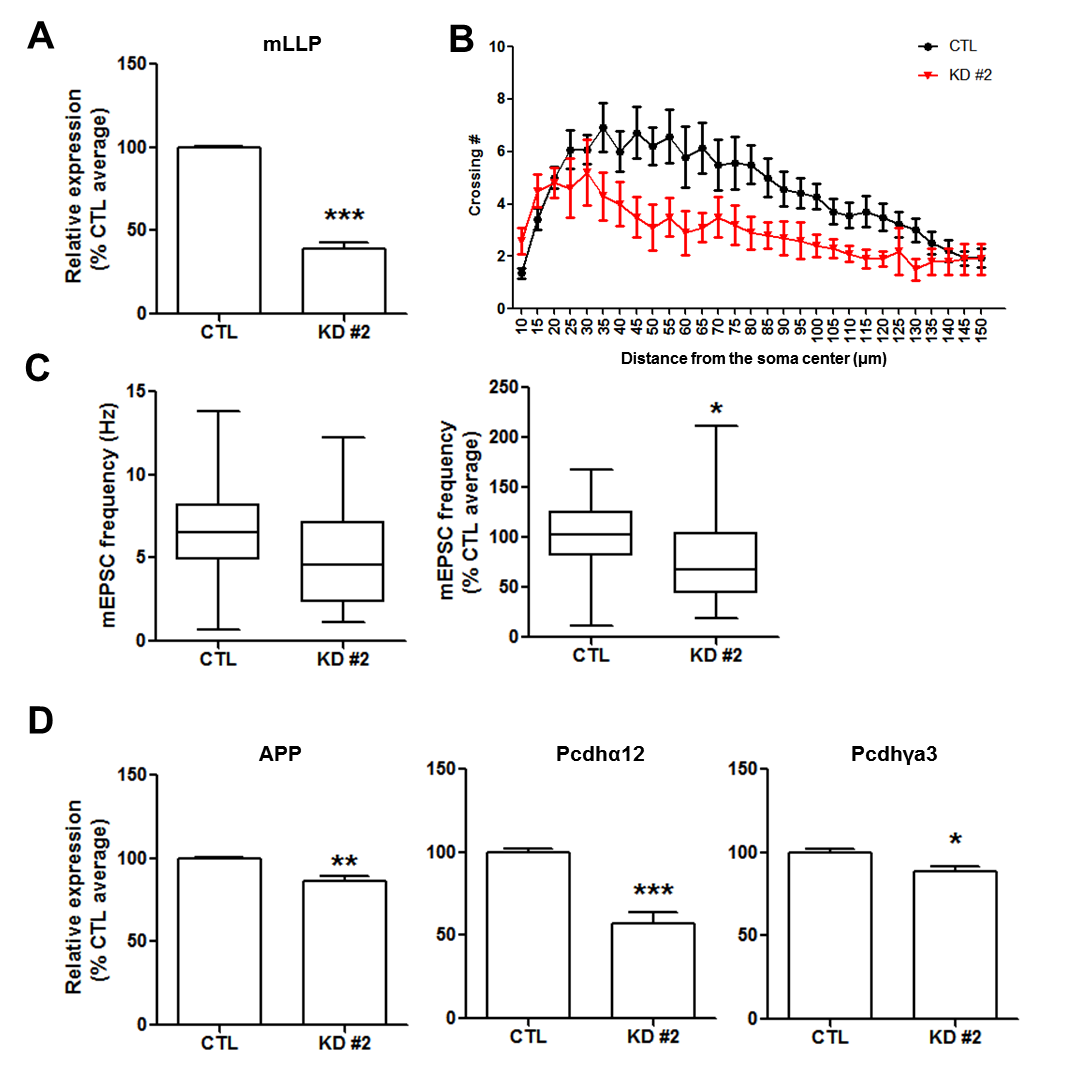


**Supplementary Figure 4. Effects of another shRNA on dendritic morphology, mEPSC frequency, and gene expression**

(A) Reduction of endogenous mLLP by ~ 61.0 % using new shRNA-expressing AAV (KD #2). Unpaired t-test: ***p < 0.0001, n = 6 per group.

(B) Sholl analysis of neurons expressing the control shRNA or KD #2 shRNA (n = 9 – 10 neurons per group). Two-way ANOVA: group effect ***p < 0.0001.

(C) (Left) mEPSC frequency of neurons expressing KD #2 shRNA at DIV 12 – 14. Mann Whitney test: p = 0.0577, n = 23 – 29 neurons per group. (Right) Because of the variation across the neuron cultures, the relative mEPSC frequency was also analyzed. It was calculated by normalizing the mEPSC frequency of each neuron by the average value of CTL neurons for each set of experiment. Mann Whitney test: *p = 0.0341.

(D) mLLP knockdown with new shRNA also affects the expression of APP and some protocadherin genes. qRT-PCR results measuring the each mRNA level normalized to GAPDH level. The relative expression level was calculated by normalizing the gene/GAPDH value by that of scrambled control (SCR) average. Unpaired t-test, ** p < 0.01, *** p < 0.001, n = 3 – 6 per group.


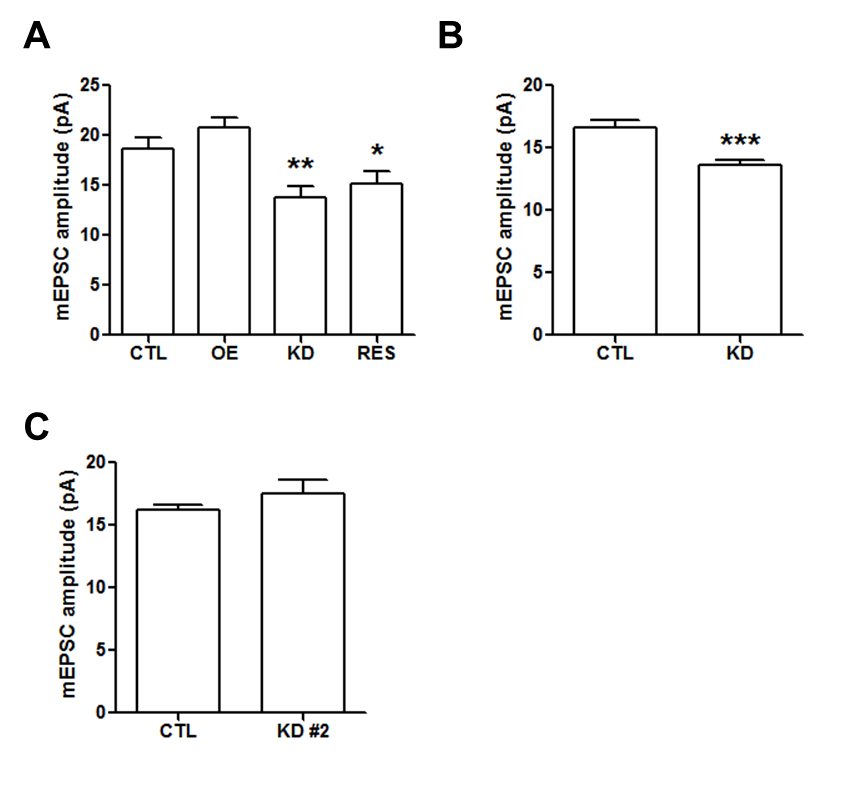


**Supplementary Figure 5. mEPSC amplitude was not affected by mLLP knockdown or overexpression despite some reduction in KD neurons**

(A) mEPSC amplitude was reduced in KD and RES neurons at DIV 10 – 11 (one-way ANOVA with *post hoc* Newman-Keuls multiple comparison Test: n = 10 – 13 neurons, KD VS CTL, **p < 0.01; RES VS CT, *p < 0.05; OE VS CTL, KD VS RES, not significant). The reduction of mEPSC amplitude was not significantly rescued in RES neurons.

(B) mEPSC amplitude was also reduced in KD neurons at DIV 12 – 14 (unpaired t-test: n = 14 – 18 neurons, ***p = 0.0006).

(C) mEPSC amplitude was not affected in KD #2 neurons at DIV 12 – 14 (unpaired t-test: n = 23 – 29 neurons, p = 0.2187)


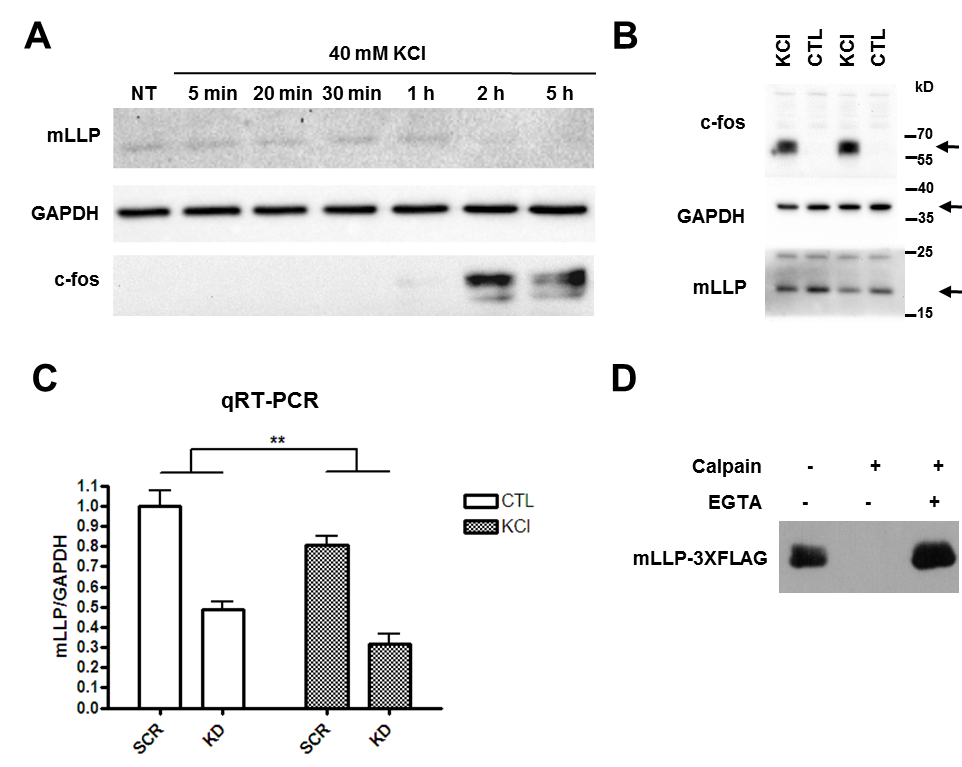


**Supplementary Figure 6. Down-regulation of mLLP by neuronal activity and calpain**

(A) Western blot analysis showing that neuronal activation by KCl at DIV 7 decreases mLLP protein expression level. NT, no treatment control.

(B) Full blot images including the data presented in Fig. 5A.

(C) qRT-PCR analysis of mLLP expression after 2 h KCl stimulation of neuron culture showed that mLLP mRNA level was reduced by approximately 20 % by neuronal activity. Significant effect of KCl was detected (Two-way ANOVA, effect of KCl, **p = 0.0084).

(D) mLLP proteolysis by calpain. Lysates of HEK293T cells expressing mLLP-3XFLAG were subjected to calpain reaction with or without EGTA, the calcium chelator. Calpain-mediated proteolysis of mLLP was dependent on calcium.

**
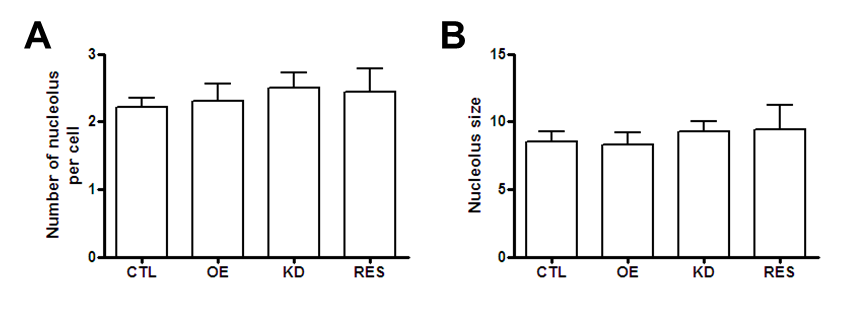
**

**Supplementary Figure 7. mLLP knockdown does not impair the gross changes of nucleolar structure**

(A-B) AAV expressing shRNA against mLLP or control shRNA were infected into the dissociated neuron culture with shRNA-resistant mLLP expressing or control vectors. Intense spots detected by fibrillarin antibody were analyzed using ImageJ. mLLP knockdown or overexpression did not affect neither number (A) nor size (B) of nucleoli (One way ANOVA, p = 0.738 for nucleolus number, p = 0.831 for nucleolus size, n = 9 – 19 cells per group).


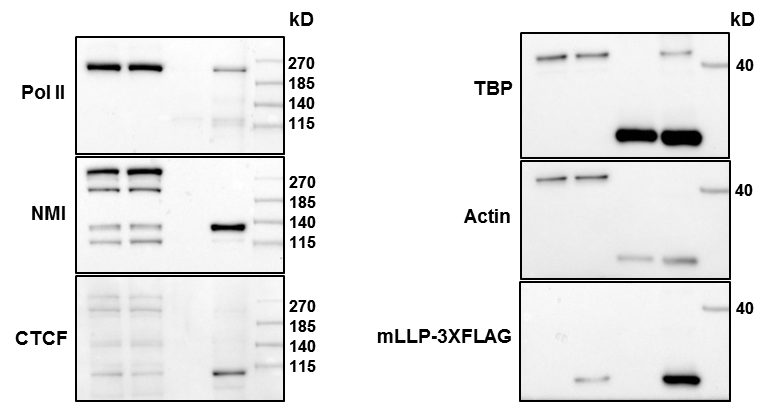


**Supplementary Figure 8. Co-immunoprecipitation of transcriptional regulators with mLLP**

Extended blot images of data represented in Fig. 5B were merged with the membrane images. Exposure times are different from those in Fig. 5B. All data were obtained on the same blot. To analyze multiple proteins on the same blot, the membrane was cropped according to the size of the protein to be detected, and different antibodies were probed after removing the previously probed antibodies by stripping buffer.


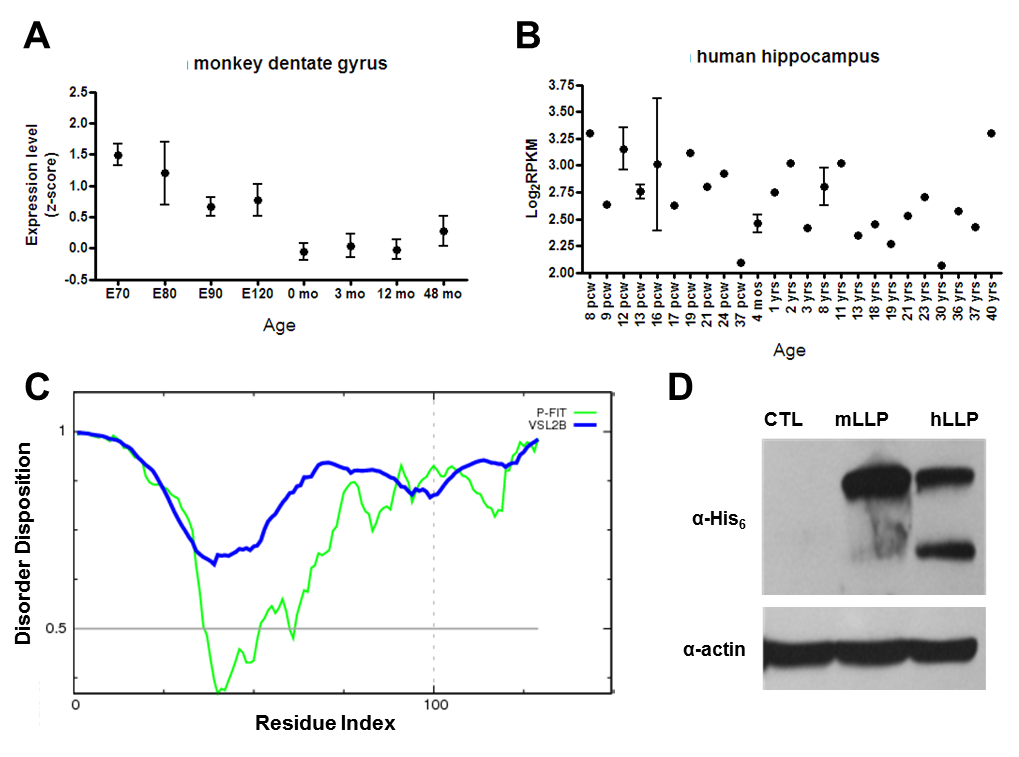


**Supplementary Figure 9. hLLP is similar with mLLP in terms of expression pattern, intrinsic disorder, and cell permeability**

(A-B) An example of the developmental monkey (A) or human (B) transcriptome data from Allen Brain Atlas was represented as a graph along the developmental time course.

(C) Disorder analysis of hLLP using VSL2B and PONDR-fit in DISPROT.

(D) hLLP protein added in the medium was detected in the cell lysate. hLLP protein purified from E.coli was partially cleaved but both the intact and cleaved forms could enter the cells.
